# Evidence of Airborne Excretion of *Pneumocystis carinii* during Infection in Immunocompetent Rats. Lung Involvement and Antibody Response

**DOI:** 10.1371/journal.pone.0062155

**Published:** 2013-04-23

**Authors:** Jean Menotti, Alexandra Emmanuel, Chafia Bouchekouk, Magali Chabe, Firas Choukri, Muriel Pottier, Claudine Sarfati, El Moukhtar Aliout, Francis Derouin

**Affiliations:** 1 Department of Parasitology-Mycology, E.A.3520, Paris-Diderot University, Sorbonne Paris Cité and Saint-Louis Hospital, Assistance Publique – Hôpitaux de Paris, Paris, France; 2 Biology and Diversity of Emerging Eukaryotic Pathogens (BDEEP), Center for Infection and Immunity of Lille (CIIL), Pasteur Institute of Lille, Inserm U1019, CNRS UMR 8204, Université Lille-Nord de France, Lille, France; Instituto de Salud Carlos III, Spain

## Abstract

To better understand the role of immunocompetent hosts in the diffusion of *Pneumocystis* in the environment, airborne shedding of *Pneumocystis carinii* in the surrounding air of experimentally infected Sprague Dawley rats was quantified by means of a real-time PCR assay, in parallel with the kinetics of *P. carinii* loads in lungs and specific serum antibody titres. *Pneumocystis*-free Sprague Dawley rats were intratracheally inoculated at day 0 (d0) and then followed for 60 days. *P. carinii* DNA was detected in lungs until d29 in two separate experiments and thereafter remained undetectable. A transient air excretion of *Pneumocystis* DNA was observed between d14 and d22 in the first experiment and between d9 and d19 in the second experiment; it was related to the peak of infection in lungs. IgM and IgG anti-*P. carinii* antibody increase preceded clearance of *P. carinii* in the lungs and cessation of airborne excretion. In rats receiving a second challenge 3 months after the first inoculation, *Pneumocystis* was only detected at a low level in the lungs of 2 of 3 rats at d2 post challenge and was never detected in air samples. Anti-*Pneumocystis* antibody determinations showed a typical secondary IgG antibody response. This study provides the first direct evidence that immunocompetent hosts can excrete *Pneumocystis* following a primary acquired infection. Lung infection was apparently controlled by the immune response since fungal burdens decreased to become undetectable as specific antibodies reached high titres in serum. This immune response was apparently protective against reinfection 3 months later.

## Introduction

*Pneumocystis* pneumonia (PCP) due to *P. jirovecii* remains a serious opportunistic infection among immunocompromised patients, mainly human immunodeficiency virus (HIV)-infected persons, haematology patients and bone marrow or organ transplant recipients [1]–[4]. Both animal and human studies are in favour of an airborne transmission of *Pneumocystis* and support the role of PCP patients and colonized hosts as potential sources of *Pneumocystis*. In animal models, this has been evidenced by co-housing experiments, showing transmission of *Pneumocystis* from animals with PCP to immunocompromised or immunocompetent recipients, but also from asymptomatic carriers to naive recipients [5]–[9]. Arguments in favour of a similar pattern of transmission of *P. jirovecii* in human are emerging, especially from the analysis of PCP cluster cases showing genotypic identities between *P. jirovecii* DNA recovered from patients with PCP [10]–[13], colonized patients [14] and colonized health care workers [15]. Several studies have found very variable prevalence rates of colonization in non immunocompromised persons [16] raising the hypothesis that *Pneumocystis* could be maintained in the general population through human-to-human transmission, with asymptomatic carriers or primarily infected patients as main reservoirs. However, information on *Pneumocystis* excretion at this stage of infection is still lacking to support this assumption.

As we previously showed that *Pneumocystis* was detectable and quantifiable in the close environment of patients or rats with PCP [17], [18], we examined the kinetics of infection and airborne excretion of *Pneumocystis* by immunocompetent rats following experimental infection and reinfection with *P. carinii*.

## Materials and Methods

### Ethics Statement

All animal experiments complied with the European Communities Amendment of Cruelty to Animals Act 1976 and the Directive 86/609/EEC on the Protection of Animals Used for Experimental and Other Scientific Purposes, updated in the Council of Europe’s Appendix A (http://conventions.coe.int/Treaty/EN/Treaties/PDF/123-Arev.pdf). The protocol was approved by the Ethical Committee for experiments on animals of the region Nord-Pas-de-Calais (approval number CEEA 022011).

### Rats

*Pneumocystis*-free Sprague Dawley (SD) rats were purchased from Charles River (L’arbresle, France). Before ordering rats for our study, we checked the *Pneumocystis*-free status on sample rats originating from two different barrier-room breeding colonies of the Charles River’s facilities, using our *in lab* immunofluorescence antibody test for *P. carinii* (IFA, see below) and *P. carinii*-specific PCR test [9]. Sixteen sample rats were examined before our first experiment, and the breeding zone in which all sample rats were negative by PCR and IFA was selected. Before the second experiment, this control was repeated on 6 sample rats from the same zone. During the experiments, no other experiment with *P. carinii* was conducted in the laboratory. All animals were housed in HEPA-filtered air isolators and were allowed sterile food and sterile water *ad libitum*. Cage cleaning and/or animal manipulations were always done in sterile conditions under a laminar flow hood.

### Infection and Experimental Protocols

*P. carinii* organisms were collected and purified from the lungs of heavily infected dexamethasone-treated rats [19]. Briefly, parasites were extracted in Dulbecco’s Modified Eagle’s Medium (DMEM; BioWhittaker, France) by agitation of lung pieces with a magnetic stirrer. The resulting homogenate was poured successively through gauze, 250 and 63 µm stainless steel filters. After centrifugation, the pellet was resuspended in a haemolytic buffered solution. *P. carinii* organisms were collected by centrifugation and then purified on a polysucrose gradient (Histopaque-1077, Sigma-Aldrich, France). *P. carinii* was quantitated on air dried smears stained with RAL-555 (Réactifs RAL, France), a rapid panoptic methanol-Giemsa-like stain. *P. carinii* was then cryopreserved by placing parasites in foetal calf serum with 10% dimethyl sulfoxide (DMSO) at −80°C in a Nalgene 1°C cryo freezing container for 4 hours. The parasite samples were then stored in liquid nitrogen. In all experiments, the inoculum consisted of 10^6^ cryopreserved *P. carinii* inoculated intratracheally following a non-surgical method, after isoflurane anaesthesia, as previously described [20].

In a first experiment, 18 SD rats were inoculated. At d8, 14, 22, 29, 41 and 61 post-inoculation, 3 randomly selected rats were pooled and placed overnight into an air sampling chamber, and then were euthanatized to quantify *Pneumocystis* in lungs by qPCR and titrate anti-*P. carinii* antibodies in serum. For control, three naïve rats originating from the same colony were euthanatized at d0.

In a second experiment, 60 rats were inoculated. Thirty were used to confirm the results of the first experiment and better define the air shedding period, by testing one pool of 3 rats at d2, 6, 9, 13, 16, 19, 22, 29, 41, and 55 post-inoculation. The remaining 30 rats were maintained in a protected environment for 3 months and then re-inoculated with 10^6^ *P. carinii* to examine the consequence of a second challenge on lung infection and air shedding. After the second challenge, 3 rats were pooled for air samples at d2, 6, 9, 11, 13, 16, 19, 22, 29, and 42 post-inoculation, and then euthanatized to collect lungs and serum.

### Air Samples

Collection of air samples in the environment air surrounding infected rats was performed as previously described [18] with minor modification. Briefly, a specific device consisting of Coriolis®µ biocollector (Bertin Technologies, Montigny, France) directly connected to a HEPA-filtered air sampling chamber was used. At each sampling date, 3 rats were maintained overnight in the sampling chamber, and then one air sample was collected during 3 min at a flow rate of 300 L/min on 15 ml of sterile PBS +0.002% Tween 80. The collection liquid was centrifuged at 2500 g for 10 min and the supernatant was removed to provide a 1-mL pellet that was stored at +4°C for later quantitative PCR assay. Samplings were performed in a positive pressure laboratory and the sampling device was placed in a laminar air flow cabinet. Before each air sample, the chamber was carefully cleaned with a chemical disinfectant (Aniospray, Laboratoires Anios, France) and a control air sample was collected.

### Assessment of Infection in Rats

After air collection, each of the 3 sampled rats was euthanatized. The whole lung was collected aseptically, and then isolation of *P. carinii* organisms from lung tissue was performed by stomaching, filtration and NH_4_Cl haemolysis, as previously described [19]. The resulting homogenate was centrifuged and the pellet was resuspended in 1 ml and then stored at +4°C for later quantitative PCR assay. Blood was collected by cardiac puncture and centrifuged; the collected serum was kept frozen until serology was performed.

### Real-time PCR

DNA extraction and PCR analysis targeting the mitochondrial large subunit rRNA gene of *P. carinii* were performed as previously described [18]. Briefly, air samples (200 µL of each pellet) and lung samples (10% v/v of each lung pellet) were analyzed in separate PCR runs to avoid any cross contamination. Ten units of lyticase (Sigma-Aldrich, Saint Quentin Fallavier, France) were first added to each of the pulmonary and air sample pellets. After mixing, the samples were incubated for 30 min at 37°C. DNA was then extracted by using the QIAamp DNA Mini Kit (Qiagen, Hilden, Germany) according to the manufacturer’s instructions. The real-time quantitative PCR assay was performed with the previously described primers and probe [18] on an Applied Biosystems 7500 PCR system in a 25-µl volume, by using the TaqMan Universal Master Mix with uracyl-DNA-glycosylase (Applied Biosystems, Foster City, CA, USA), with 0.4 µM each primer, 0.2 µM probe, and 5 µl of extracted DNA sample. After 2 min at 50°C and 10 min at 95°C, amplification consisted of 45 cycles of 15 s of denaturation at 95°C, followed by 1 min of annealing and extension at 60°C. All samples were tested in duplicate. The detection limit of the assay was 1 *P. carinii* organism/mL. Each PCR run comprised a calibration set consisting of seven serial ten-fold dilutions of a *P. carinii* suspension, ranging from 1 to 10^6^ *P. carinii* organisms/mL. The number of *P. carinii* organisms in air and lung samples were extrapolated from the Ct values, then adjusted to the original sample size for a final expression of the results in number of *P. carinii* organisms/air sample or/lung.

### Anti-*Pneumocystis carinii* Antibody Determination

Anti-*Pneumocystis* antibody (IgM, IgG) titres were determined by an indirect fluorescence antibody assay (IFA) using slides coated with *P. carinii*. Serial dilutions of sera (pure to 1∶12 000) were prepared in phosphate-buffered saline (PBS) and then incubated on *P. carinii* slides. Alexa 488 goat anti-rat IgM (µ chain) or IgG (H+L) was used as antibody conjugate (Invitrogen). Sera and conjugates were incubated at 37°C in a moist chamber for 30 min, and slides were rinsed and washed with PBS after each incubation period (5 min.×2). Positive and negative control serum samples were run with each assay. The antibody titre was the last dilution that shows fluorescence.

### Statistical Analysis

Fisher’s exact test was performed using Graphpad Prism v.5.01 for Windows (Graphpad Software, San Diego, CA, USA, www.graphpad.com) to analyze the relationship between IgG titres and lung burdens.

## Results

### Primary Infection

*Pneumocystis* was never detected in the lungs and surrounding air of the control rats. In addition, *Pneumocystis* was never detected in any of the control air samples performed during the experiments.

**Figure 1:**
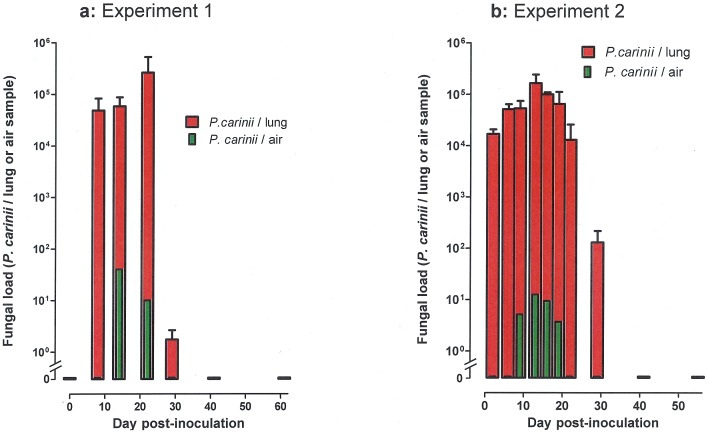
Kinetics of *Pneumocystis carinii* air shedding and lung burden in Sprague Dawley rats inoculated intratracheally at day 0 with 10^6^ cryopreserved *P. carinii* organisms. **Figure 1a**: experiment 1; **Figure 1b**: experiment 2. In each experiment, the total number of *P. carinii* organisms was determined by qPCR in lung samples (red bars) and in air samples (green bars).

**Figure 2:**
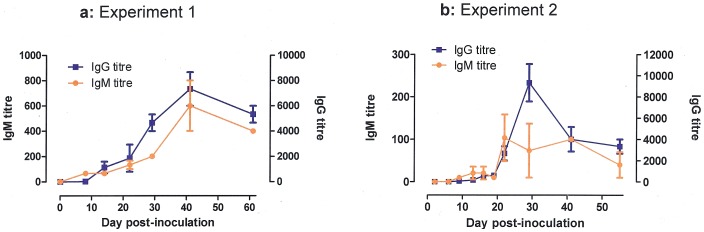
Kinetics of anti-*Pneumocystis* antibody titres in Sprague Dawley rats inoculated intratracheally at day 0 with 10^6^ cryopreserved *P. carinii* organisms. **Figure 2a**: experiment 1; **Figure 2b**: experiment 2. In each experiment, anti-*Pneumocystis* IgG (blue squares) and IgM (orange circles) antibody titres were determined by an indirect immunofluorescence assay.

In the first experiment, *Pneumocystis* was detected in the lungs of the infected 3 rats examined at d8, d14, d22, with mean fungal burdens of 48,600, 58,300 and 262,800 *P. carinii* organisms/lung respectively. At d29, 2 of 3 rats were still PCR positive, with a mean fungal burden of 2.5 *P. carinii* organisms/lung. Thereafter, all rats were PCR negative (**Figure 1a**). *P. carinii* DNA was detected in the air samples collected at d14 and d22 (40 and 10 *P. carinii* organisms/air sample, respectively), but not at d8 and from d29. Serum anti-*Pneumocystis* antibodies were detectable from d8 for IgM and d14 for IgG. For both isotypes, titres increased until d41 with a mean approximate peak value of 1/600 for IgM and 1/7000 for IgG (**Figure 2a**).

The second experiment confirmed and extended the results of the first experiment (**Figures 1b** **and** **2b**). The closer follow-up of inoculated rats allowed showing a progression of infection within the first two weeks, as assessed by almost 1 log increase of fungal burdens in lungs from day 6 to day 13. Fungal burdens remained over 10^4^ *Pneumocystis*/lung until d19, and then decreased at d22 and d29 to become undetectable at d41 and thereafter. *Pneumocystis* was detected in the air at d9, d13, d16, and d19 with fungal loads that grossly followed the kinetics of fungal burdens in lungs. As in the first experiment, IgM and IgG anti-*Pneumocystis* antibodies were detectable from d9 and d13 respectively, then sharply increased till d22 and d29, respectively, to remain at a high level until the end of the experiment. In both experiments, the increase of antibody titres was apparently related with the clearance of *Pneumocystis* in lung and cessation of air shedding. Data plotting of IgG titres with lung burdens showed that an IgG antibody titre >1/3000 was significantly associated with a low (<100 *P. carinii* organisms/lung) or undetectable fungal burden in lungs (P<0.0001 and P = 0.0011 for experiments 1 and 2, respectively).

### Reinfection

In rats receiving a second challenge by 10^6^ *Pneumocystis* 3 months after the first inoculation, *Pneumocystis* was only detected at a very low level in the lungs of 2 of 3 rats at d2 post challenge (8.0 and 8.4 *P. carinii* organisms/lung respectively) and was never detected thereafter (data not shown). No *P. carinii* organism was detected by qPCR in any of the 10 collected air samples. Anti-*Pneumocystis* antibody determinations showed a typical secondary antibody response, with a marked increase of the mean IgG antibody titre from 1/6000 at the time of challenge to 1/13000 11 days later.

## Discussion

This study provides the first direct evidence that an immunocompetent host can excrete *Pneumocystis* following a primary acquired infection. This was reproducibly demonstrated in two separate experiments performed in Sprague Dawley rats infected intratracheally by *P. carinii*. In both experiments, airborne excretion was transient and concomitant to the peak level of infection in lungs. Indeed, one can argue that this observation was made in experimental conditions, but should acknowledge some great similarities between the kinetics and levels of fungal burden in lung and antibody responses in our model and in naturally infected rats [21], despite a longer delay to reach high fungal burden in natural conditions. Interestingly, in both natural and experimental conditions, lung infection was apparently controlled by the immune response since fungal burdens decreased to become undetectable as specific antibodies reached high titres in the serum [7], [21], [22]. Our results show that immunity apparently resulted in the control of air shedding.

The airborne transmission of *Pneumocystis* has been suspected for many years but the true conditions of transmission between immunocompetent hosts were unclear till now as the excretion of *Pneumocystis* was not characterized. The finding of a transient air excretion of *Pneumocystis* at the early phase of primary infection contributes to elucidate this transmission and the maintenance of *P. carinii* in rat colonies. Recently, two studies have established a close link between primary *P. carinii* infection and interstitial pneumonia in immunocompetent rats [21], [23]. Other species of *Pneumocystis* are likely to induce a comparable pathology in other animal species, as already demonstrated in rabbits which develop a spontaneous pneumocystosis at weaning [24], [25], in young piglets [26] and possibly in humans, in whom *Pneumocystis* primary infection occurs in about 95% of immunocompetent 2–4-year-old children [27], [28]. Although this primary infection could usually remain subclinical, such hypothesis seems conceivable, considering the occurrence of *Pneumocystis*-related bronchiolitis and upper tract respiratory symptoms in immunocompetent children [28], [29] or reports indicating that *Pneumocystis* primary infection can present clinically as pneumonia in immunocompetent infants [30]. If so, air shedding of *Pneumocystis* could frequently occur in the general population and support the circulation and maintenance of *Pneumocystis* in the absence of environmental reservoir. The low level of air shedding during primary infection, the control of infection and air shedding by immunity, as well as the resistance to reinfection that we found in the rat model and that was previously demonstrated in a mouse model [8], could be limiting factors for this diffusion. Consistently, the increasing prevalence of antibody titres during infancy and the high antibody titres found in health care workers potentially exposed to *Pneumocystis* [31], [32] suggest a control of *Pneumocystis* spread in humans. However, it remains crucial to better document *P. jirovecii* excretion by colonized patients and healthy individuals in order to refine preventive measures for naïve immunocompetent hosts and immunocompromised patients.
