# Correction: Evidence of Airborne Excretion of *Pneumocystis carinii* during Infection in Immunocompetent Rats. Lung Involvement and Antibody Response

**DOI:** 10.1371/annotation/f1ecc4cc-fc10-4605-98e5-181adf4cb325

**Published:** 2013-06-27

**Authors:** Jean Menotti, Alexandra Emmanuel, Chafia Bouchekouk, Magali Chabe, Firas Choukri, Muriel Pottier, Claudine Sarfati, El Moukhtar Aliout, Francis Derouin

The name of the eighth author was spelled incorrectly. The correct name is: El Moukhtar Aliouat. 

